# Uterine leiomyoma in pediatric population: A case report and review of the literature

**DOI:** 10.3389/fped.2022.1020072

**Published:** 2022-10-04

**Authors:** Cristina Martucci, Alessandro Crocoli, Giorgio Persano, Marco Bonito, Alessandra Stracuzzi, Rita Alaggio, Arianna Bertocchini, Antonella Accinni, Alessandro Inserra

**Affiliations:** ^1^ General Surgery Unit, Department of Surgery, Bambino Gesù Children’s Hospital – IRCCS, Rome, Italy; ^2^ Surgical Oncology Unit, Department of Surgery, Bambino Gesù Children’s Hospital – IRCCS, Rome, Italy; ^3^ Division of Obstetrics and Gynecology, San Pietro Hospital, Rome, Italy; ^4^ Department of Pathology, Bambino Gesù Children's Hospital – IRCCS, Rome, Italy

**Keywords:** leiomyoma, uterine cancer, surgery, gynecology, pediatrics

## Abstract

Uterine leiomyomas are rare in the pediatric population with less than 20 cases in adolescences reported in the literature. Furthermore, these masses represent a common presentation of gynecologic tumors with increasing age. We report a case of a 14-year-old female who presented with abdominal pain and increasing abdominal girth. Workup with ultrasound, CT and MRI demonstrated a large pelvic mass. Complete resection by median laparotomy was performed. The mass weighed 5,596 g and was 29.5 cm × 27 cm × 19 cm; the pathological examination confirmed the hypothesis of leiomyoma. The patient remained asymptomatic at 3 months follow up.

## Introduction

Uterine leiomyomas are frequent benign tumors in adult women and they represent the most common indication for hysterectomy. However, leiomyomas are extremely rare at pediatric age, with less than 15 adolescent cases reported in the literature (1–11). We report a rare case of a uterine leiomyoma in an adolescent presenting as a 25 cm pelvic mass and discuss the frequency, etiology, and fertility consequences of uterine leiomyomas in this specific population.

## Case description

A 14-year-old Caucasian gravida 0 presented to the emergency department complaining of abdominal pain and increasing abdominal girth. Abdominal exam revealed a soft abdomen with a voluminous, partially mobile, pelvic mass extending to the level of the xiphoid process.

Pelvic ultrasound revealed a voluminous mass, displacing the uterus and the ovaries and measuring approximately 20 cm. Subsequently, the patient underwent Computed Tomography (CT) scan, which confirmed the presence of an abdominal solid lesion (with size 25 cm × 18 cm), intensively vascularized, with multiple areas of impaired signals, ranging from the pelvis up to the right hypochondrium; the mass made contact with the fundus of the uterus and caused pelvic varicocele.

After multidisciplinary meeting, the patient underwent percutaneous biopsy of the tumor, which showed a proliferation of bland and homogeneous spindle cells without atypia or mitosis. The cells were positive for smooth muscle actin and negative for HMB45. Considering the size of the lesion, only a morphological diagnosis of spindle cell neoplasm was rendered.

Magnetic Resonance Imaging (MRI) was then performed in order to better characterized tumor's contact with nearby structures: the lesion, widely inhomogeneous, presented a large central areas inhomogeneously hypointense in the T2-weighted sequences and showed an inhomogeneous post-contrast enhancement, more evident at the periphery, and inhomogeneous restriction of diffusivity. The lower portion tightly adhered to the anterior portion of the uterine fundus, without a clear cleavage plan. The mass contacted superiorly the liver, anteriorly the rectus abdominals and posteriorly the spine (causing marked compression on the inferior vena cava); the ovaries, located medially and characterized by the presence of multiple follicles, were not involved by the tumor. Tumor markers, including Beta-human chorionic gonadotropin (β-HCG), Alfa-fetoprotein (α-FP), CA 19-9, CA-125 and CEA, were within normal limits.

**Figure 1:**
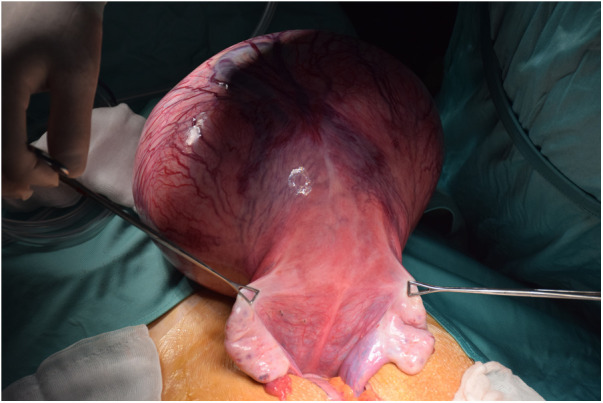
Intraoperative image showing the proximity of the lesion to the fallopian tubes and ovaries.

**Figure 2:**
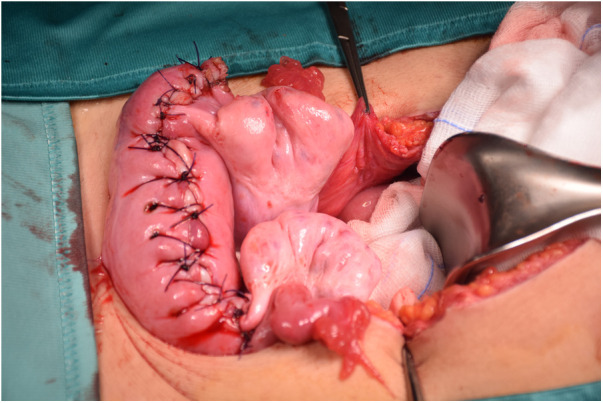
Intraoperative image showing the suture of the fundus and the final aspect of the uterus.

After multidisciplinary meeting, surgical excision of the mass was indicated. Median laparotomy was performed; upon opening, the abdomen was widely occupied from the lesion, arising from the fondus of the uterus. The left and right ovaries were inspected and found to be grossly normal (Figure 1). No pathological lymph nodes or peritoneal implants were present. “En-bloc” resection of the mass was accomplished, but the encasement of the uterine fondus didn't allow to preserve the entrance of the fallopian tubes. The uterine wall was then was reapproximated by double interrupted stitches in adsorbable suture (Figure 2).

**Figure 3:**
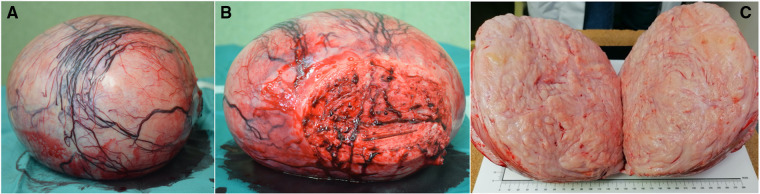
Images of the specimen: (**A**) lateral view; (**B**) caudal view, including part of the uterine fundus; (**C**) intralesional sagittal view.

**Figure 4:**
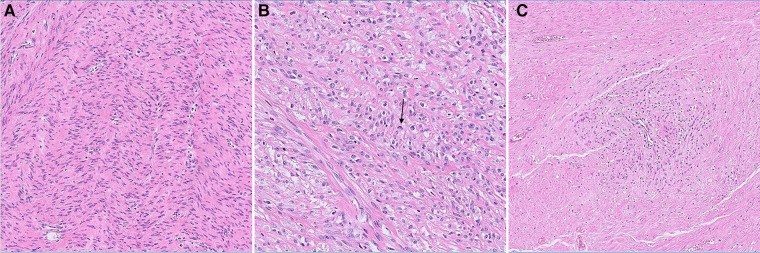
(**A**) interlacing bundles of spindle cells proliferation with (**B**) focus of enlarged nuclei, rare mitotic figures (black arrow) and (**C**) small areas of ischemic necrosis with viable perivascular elements.

Grossly the resected specimen weighed 5,596 g (Figure 3) and measured 29.5 cm × 27 cm × 19 cm. The cross-sectional surface showed a whitish color and a translucent appearance. We performed an extensive sampling, that confirmed a spindle cell neoplasm consisting of loosely arranged bundles of elements with none to mild atypia. Rare, typical mitoses were present (<5/10 HPF). We also identified small foci of ischemic-type necrosis with viable cells around vessels. Based on the WHO 2020 classification of Female genital Tumors, a definitive diagnosis of leiomyoma was achieved (Figure 4).

She did well postoperatively; the patient presented regular, painless menstrual cycles. Follow-up ultrasound three months later showed no recurrence of the lesion.

## Discussion

Uterine leiomyomas are benign tumors that arise from the uterus' smooth muscle cells and extracellular matrix. Leiomyomas are reported in 4%–11% of the female population, and their prevalence rises with age, reaching approximately 40% by the age of 50 (5, 9). These tumors are rarely symptomatic in teenagers and are almost unreported in prepubescent children, although there have been two autopsy reports of uterine leiomyomas in a 1-year-old child and an 8-year-old child (3, 12). African American race, nulliparity, family history, significant alcohol use and red meat intake are all connected with an increased incidence of leiomyomas (4, 13, 14). Fibroids appear to be reduced by smoking and oral contraceptive pills (OCPs) (12, 14). Although the exact cause of fibroids is unknown, hormones and genetics are known to play a role in their genesis, growth, and regression.

Pediatric patients with leiomyoma typically present with significant symptoms, such as abdominal pain or distensions, back pain, irregular vaginal bleeding or urine retention. These tumors produce symptoms referable to their size and location: the submucous lesions produce metrorrhagia (due to endometrial ulceration), the intramural ones result in menorrhagia (because the interfere with myometrial contraction), and the subserosal ones usually remain asymptomatic. They may also become large enough to block the ureters, interfere with pregnancy, or cause inflammatory complications. Occasionally, the initial clinical scenario may include life-threatening conditions such as uremia and hemorrhagic shock (1–11, 15). The diagnosis was usually made with a pelvic exam and ultrasound. The dimension of the leiomyomas described in pediatric population ranges from 7 cm to 30 cm; the majority were large enough to be palpated during an abdominal/pelvic exam and large enough to produce discomfort due to mass impact.

Leiomyomas are benign masses arising, subserosally, intramurally or submucosally, from the smooth muscle of the uterus and enclosed by a thin pseudocapsule of areolar tissue, according to pathological investigation. Leiomyomas displace and compress surrounding structures rather than invading them. There are histological features in the teenage population that appear to signal malignancy: enhanced cellularity, increased mitotic activity, and cellular atypia. Malignant transformation is a concern in this population due to these histological traits, fast growth, and the very large size of the leiomyomas themselves. In the first description of a leiomyoma in a teenage girl, Wisot documented a 13-year-old girl who appeared with irregular vaginal bleeding and a growing abdominal mass and was found to have a 12 cm uterine leiomyoma (11). A uterine myoma with cystic degeneration was discovered on histological analysis, however sarcoma was initially suspected. In another case report by de Rooy (5), a 15-year-old girl with a 16.5 cm tumor presented with hemorrhagic shock due to a ruptured uterine artery. Because of enhanced mitotic figures, histological investigation suggested benign cellular leiomyoma rather than low-grade leiomyosarcoma. The combination of this histological finding and the tumor's expansive growth was concerning, but the ultimate pathology diagnosis was benign uterine leiomyoma. Morad described a 15-year-old girl who underwent myomectomy for a symptomatic 7 cm uterine tumor seven years later (9). A tumor with high cellularity, vascular invasion, and tiny deposits of smooth muscle cells within vascular channels were discovered on histological inspection. Although the clinical and histological appearance made differential diagnosis challenging, the tumor was judged to be a leiomyoma with vascular intrusions rather than a low-grade endometrial sarcoma. Our patient's pathological findings were spindle cell neoplasm consisting of loosely arranged bundles of elements with none to mild atypia. Rare, typical mitoses were present (<5/10 HPF) and small foci of ischemic-type necrosis with viable cells around vessels were identified.

Leiomyomas are treated with observation, medical therapy, and/or surgery. Observation may be adequate if the leiomyoma is asymptomatic or medically managed with hormone therapy. Medical treatment with progestational drugs has been reported in the literature to reduce myoma growth and control hemorrhage; however, well-documented controlled studies confirming their effectiveness are missing (16). The one patient handled non-operatively out of the previously reported pediatric cases was a 15-year-old female who arrived with umbilical pain at 22 weeks of pregnancy and was diagnosed with a fundal leiomyoma (6). All of the other patients received myomectomy for symptomatic lesions and none of them presented recurrence after long-term follow-up. Surgery is generally recommended for recurring or persistent symptoms, as well as when malignancy or recurrent spontaneous abortions are a concern. Surgical removal of a symptomatic leiomyoma is usually the therapy of choice in adolescent patients; it usually consisting in a myomectomy, which preserves fertility while eliminating the symptomatic fibroid. In our case, since it was impossible to distinguish the neoplasm from healthy uterine tissue, it was necessary to perform an “en bloc” excision of the uterine fundus, preserving the fallopian tubes.

Follow-up is crucial, especially in situations where histology was doubtful, to watch for symptom relief and probable regrowth. These patients should have their symptoms resolved while maintaining their fertility with correct surgical technique and follow-up. In the presented case, the surgical excision of the lesion involving the fallopian tubes entry may necessitate tubal patency testing in the future to confirm the likelihood of spontaneous pregnancy or to indicate medically aided procreation.

**Table 1:** Timeframe of the relevant events

**Time**	**14th March 2022**	**16th March 2022**	**22nd March 2022**	**20th April 2022**	**3rd May 2022**	**1st August 2022**
**Event**	Access to emergency department complaining of abdominal pain and increasing abdominal girth	CT scan	Percutaneous biopsy	Abdominal MRI	Surgical excision	Last follow-up

## Data Availability

The original contributions presented in the study are included in the article/Supplementary Material, further inquiries can be directed to the corresponding author/s.
